# Platelet-derived growth factor predicts prolonged relapse-free period in multiple sclerosis

**DOI:** 10.1186/s12974-018-1150-4

**Published:** 2018-04-14

**Authors:** Mario Stampanoni Bassi, Ennio Iezzi, Girolama A. Marfia, Ilaria Simonelli, Alessandra Musella, Georgia Mandolesi, Diego Fresegna, Patrizio Pasqualetti, Roberto Furlan, Annamaria Finardi, Giorgia Mataluni, Doriana Landi, Luana Gilio, Diego Centonze, Fabio Buttari

**Affiliations:** 1 Unit of Neurology and Unit of Neurorehabilitation, IRCCS Istituto Neurologico Mediterraneo (INM) Neuromed, Via Atinense 18, 86077 Pozzilli, IS Italy; 2 Multiple Sclerosis Research Unit, Department of Systems Medicine, Tor Vergata University, Via Montpellier 1, 00133 Rome, Italy; 3 Service of Medical Statistics & Information Technology, Fondazione Fatebenefratelli per la Ricerca e la Formazione Sanitaria e Sociale, Lungotevere de’ Cenci 5, 00186 Rome, Italy; 4 University and IRCCS San Raffaele, Via di Val Cannuta, 247, 00166 Rome, Italy; 5 Division of Neuroscience, Institute of Experimental Neurology, San Raffaele Scientific Institute, Via Olgettina 58, 20132 Milan, Italy

**Keywords:** PDGF, RR-multiple sclerosis, CIS, Neuroinflammation, Cytokines

## Abstract

**Background:**

In the early phases of relapsing-remitting multiple sclerosis (RR-MS), a clear correlation between brain lesion load and clinical disability is often lacking, originating the so-called clinico-radiological paradox. Different factors may contribute to such discrepancy. In particular, synaptic plasticity may reduce the clinical expression of brain damage producing enduring enhancement of synaptic strength largely dependent on neurotrophin-induced protein synthesis. Cytokines released by the immune cells during acute inflammation can alter synaptic transmission and plasticity possibly influencing the clinical course of MS. In addition, immune cells may promote brain repair during the post-acute phases, by secreting different growth factors involved in neuronal and oligodendroglial cell survival. Platelet-derived growth factor (PDGF) is a neurotrophic factor that could be particularly involved in clinical recovery. Indeed, PDGF promotes long-term potentiation of synaptic activity in vitro and in MS and could therefore represent a key factor improving the clinical compensation of new brain lesions. The aim of the present study is to explore whether cerebrospinal fluid (CSF) PDGF concentrations at the time of diagnosis may influence the clinical course of RR-MS.

**Methods:**

At the time of diagnosis, we measured in 100 consecutive early MS patients the CSF concentrations of PDGF, of the main pro- and anti-inflammatory cytokines, and of reliable markers of neuronal damage. Clinical and radiological parameters of disease activity were prospectively collected during follow-up.

**Results:**

CSF PDGF levels were positively correlated with prolonged relapse-free survival. Radiological markers of disease activity, biochemical markers of neuronal damage, and clinical parameters of disease progression were instead not influenced by PDGF concentrations. Higher CSF PDGF levels were associated with an anti-inflammatory milieu within the central nervous system.

**Conclusions:**

Our results suggest that PDGF could promote a more prolonged relapse-free period during the course of RR-MS, without influencing inflammation reactivation and inflammation-driven neuronal damage and likely enhancing adaptive plasticity.

## Background

Multiple sclerosis (MS) is an immune-mediated inflammatory disorder of the central nervous system (CNS). The majority of MS patients show a relapsing-remitting (RR) disease course, characterized by clinical stability periods of variable duration alternating with acute exacerbations. Magnetic resonance imaging (MRI) may reveal disease activity also during the stable clinical phases, as new demyelinating lesions could appear even in asymptomatic patients. Furthermore, in early phases of MS, it is often difficult to demonstrate a clear association between lesion load, site, and clinical disability contributing to the so-called clinico-radiological paradox [1]. Such discrepancies may arise from different mechanisms, including synaptic plasticity which is able to reduce the clinical expression of brain damage reestablishing the excitability of neurons deprived of their synaptic inputs.

During the acute inflammatory phases of disease, infiltrating T lymphocytes and activated microglia release a number of inflammatory cytokines regulating the function of other immune cells and inducing oligodendrocyte and neuronal damage [2]. Moreover, cytokines can alter synaptic transmission [3, 4] and plasticity possibly influencing the clinical course of the disease [5, 6].

Immune cells may also promote brain repair during the post-acute phases, by secreting different growth factors involved in neuronal and oligodendroglial cell survival [7, 8]. Particularly, the platelet-derived growth factor (PDGF) is recently emerging as a key molecule possibly involved in MS clinical recovery. Indeed, PDGF stimulates neuronal differentiation [9, 10], remyelination, and oligodendrocyte density during acute inflammation [11, 12] and reduces apoptosis after chronic demyelination [13]. In addition, PDGF enhances synaptic long-term potentiation (LTP) induction in vitro [14], likely promoting recovery after brain damage. LTP is a well-characterized form of synaptic plasticity, consisting in long-lasting increase of synaptic efficacy followed by structural rearrangements mainly dependent on neurotrophin-induced protein synthesis. It has been shown that also in MS, LTP-like plasticity could be influenced by PDGF. In particular, higher PDGF concentrations in the CSF were associated to enhance LTP-like plasticity [15] and better clinical recovery after a relapse [16]. These results agree with the hypothesis that, in MS, PDGF may promote clinical compensation of new brain lesions.

On these premises, we aimed to explore whether CSF PDGF concentrations at the time of diagnosis influence the clinical course of disease in MS. In particular, higher CSF levels of this neurotrophin could minimize the expression of brain damage favoring a stable clinical course.

## Methods

### Patients

A group of 100 MS patients was included in the study. All patients were admitted to the neurological clinic of the University Hospital Tor Vergata, Rome, in the suspect of inflammatory demyelinating CNS disease. During the hospitalization, patients were diagnosed as clinically isolated syndrome (CIS) (*n* = 31) or RR-MS (*n* = 69), based on clinical, laboratory, and MRI parameters, according to published criteria [17]. Patients with CIS showed a first clinical presentation of a disease suggestive of inflammatory demyelination, compatible with MS but not fulfilling criteria of dissemination in time [18].

Demographic and clinical data were obtained from medical records. Disease onset was defined as the first episode of focal neurological dysfunction suggestive of MS. Disease duration was calculated as the number of months from disease onset to the time of hospitalization. Clinical relapses were defined as the development of new or recurrent neurological symptoms not associated with fever or infection lasting at least 24 h.

No patient was treated with immunoactive drugs before hospitalization, and corticosteroids or immunoactive therapies were initiated later. Disease-modifying therapy (DMT) was started after the confirmed diagnosis. First-line therapies included glatiramer acetate, interferon (IFN) β-1 (44 μg three times a week, 250 μg once a day, 30 μg intramuscularly), teriflunomide, or dimethylfumarate. In patients who experienced at least two relapses during 1 year of first-line DMT, escalation to second-line treatments, including natalizumab, fingolimod, and mitoxantrone, was performed.

## Study protocol

All patients underwent clinical evaluation, brain and spine MRI, and CSF withdrawal at the time of diagnosis, during hospitalization. Follow-up consisted in both neurological examination and MRI scans performed every 6 months after diagnosis. Unscheduled clinical evaluation and MRI were performed in the suspect of clinical relapses.

### Clinical evaluation

Disability was assessed by certified examining neurologist using Expanded Disability Status Scale (EDSS) [19]. Worsening of the EDSS was defined as a change from baseline of at least 1 point (for baseline EDSS ≥1) or > 1.5 points (for baseline EDSS = 0).

Two measures of disease severity were calculated combining the EDSS score with disease duration: the progression index (PI) and the multiple sclerosis severity scale (MSSS). The PI is defined as the ratio between the EDSS score and disease duration. The MSSS is an algorithm that relates the EDSS scores to the distribution of disability in patients with comparable disease durations [20].

The Bayesian Risk Estimate for Multiple Sclerosis (BREMS) score was calculated for each patient to assess the individual risk of secondary progression [21].

### CSF collection and analysis

Lumbar puncture was performed at the time of diagnosis, during hospitalization at the Neurology Department of Tor Vergata University Hospital. CSF was centrifuged and immediately stored at − 80 °C until analyzed using a Bio-Plex multiplex cytokine assay (Bio-Rad Laboratories, Hercules, CA, USA) according to the manufacturer’s instructions.

CSF concentrations of PDGF, main proinflammatory cytokines, and anti-inflammatory molecules were calculated according to a standard curve generated for the specific target and expressed as picograms per millilitre. When the concentrations were below the detection threshold, they were indicated as not detected. Assay sensitivity was as follows: PDGF (2.9 pg/ml), interleukin (IL)-12 (3.5 pg/ml), granulocyte macrophage colony-stimulating factor (GMCSF) (2.2 pg/ml), fibroblast growth factor (FGF) basic (1.9 pg/ml), granulocyte colony-stimulating factor (GCSF) (1.7 pg/ml), IL-10 (0.3 pg/ml), IL-1-receptor antagonist (ra) (5.5 pg/ml), IL-8 (1.0 pg/ml), IL-6 (2.6 pg/ml), IL-1β (0.6 pg/ml), IL-2 (1.6 pg/ml), tumor necrosis factor-alpha (TNFα) (6 pg/ml), and IFNγ (6.4 pg/ml).

The CSF levels of amyloid-β 1–42, total tau protein, and neurofilament light (NFL) protein, biomarkers of neurodegeneration and neuronal damage, were also measured. For the analysis of amyloid-β 1–42 and total tau concentrations standard procedures, using commercially available sandwich enzyme-linked immunosorbent assays (ELISA) (Innotest b-Amyloid 1–42, Innotest h-t Ag, Innogenetics, Ghent, Belgium) was employed [22]. CSF samples were dispensed into corresponding 96-well ELISA plates, pre-coated either with the monoclonal antibody 21F12 for amyloid-β 1–42 or AT120 for total tau, and incubated, respectively, with the biotinylated antibody 3D6 or HT7. Bound antibodies were then detected by a peroxidase-labeled streptavidin, after addition of a substrate solution. The reaction was stopped by sulfuric acid. The absorbance of the reaction product was read at 450 nm. The biomarker concentrations in the samples were calculated based on the amyloid-β 1–42 and tau standard sigmoid curve equation. The levels of NFL protein in CSF were measured by fitting data to a four-parameter standard curve using GraphPad Prism Software Package (San Diego, CA, USA).

### MRI

MRI examination (1,5 Tesla) consisted of dual-echo proton density, fast fluid-attenuated inversion recovery, T2-weighted spin-echo images, and pre-contrast and post-contrast T1 weighted spin-echo images. The presence of gadolinium (0.2 ml/kg i.v.) enhancing (Gd+) lesions was evaluated by a neuroradiologist who was unaware of the patient’s clinical details. A new Gd+ lesion was defined as a typical area of hyperintense signaling on post-contrast T1-weighted images. An active MRI was defined as one showing new or enlarging T2 lesions and/or post-contrast enhanced T1-weighted lesion.

### Statistical analysis

Normality distribution of continuous variables was assessed by Shapiro-Wilk test. Data were expressed as mean (standard deviation, SD) or, when necessary, median (interquartile range, IQR). Categorical variables were shown as absolute (*n*) and relative frequency (%). Spearman’s non-parametric correlation was applied to evaluate possible association between PDGF with age, clinical continuous variables, and with the levels of the main proinflammatory and anti-inflammatory molecules. The relation between two variables was depicted by a scatter plot. Association between categorical variables was examined applying chi-square or, when necessary, Fisher exact test. Difference in continuous variables between the PDGF groups was evaluated using non-parametric Kruskal–Wallis test. Mann–Whitney test was applied for non-parametric post-hoc comparisons.

Kaplan–Meier technique was applied to estimate the relapse-free survival (RFS), defined as the probability to not reach a clinical relapse, or the first MRI progression. The estimated survival and corresponding standard error (SE), the estimated mean survival times, and the corresponding SE were reported. Cox proportional hazard model [23] was applied to test the effect of demographic, clinical, and PDGF groups and cytokines on these survival times. From univariable analysis, the most significant factors based on the Wald test statistic were included in the multivariate model. Univariable significance was set at *p* ≤ 0.10. A forward selection procedure was applied to individuate the best significant predictors. All multivariable Cox regression analyses were adjusted for patients’ escalation to second line and age. To show the effect of the PDGF groups for each MRI progression levels (No or Yes), the multivariate final Cox model stratified for MRI progression was performed. The proportional hazards assumption required by the Cox model was investigated using Schoenfeld residuals. The estimated effect was shown as hazard ratio (HR) and the corresponding 95% confidence interval (95% CI). Boostrap internal validation was performed. For each group of 200 bootstrap samples, the model was refitted and tested against the observed sample in order to derive an estimate of the predictive accuracy. To evaluate the predictive accuracy, two aspects, calibration and discrimination, were used. Calibration was obtained in the form of the shrinkage coefficient to quantify the over fitting of the model. Discrimination was summarized by the c index, the probability of concordance between the predicted and the observed survival. For multiple testing, the Benjamini–Hochberg false discovery rate (FDR) controlling procedure was applied. A *p* value ≤ 0.05 was considered statistically significant. All analyses were performed using IBM SPSS Statistics for Windows (IBM Corp., Armonk, NY, USA). Model validation was performed using the rms package in R 3.4.3.

## Results

### Clinical characteristics and CSF PDGF levels in the whole patient population

In a group of 100 early MS patients, classified at the time of hospitalization as CIS or RR-MS, we explored the correlation between CSF PDGF concentrations measured at the time of diagnosis and prospective disease activity.

The median follow-up duration was 61 months, from a minimum of 2 to a maximum of 88 months.

Clinical and demographic characteristics of MS patients are shown in Table 1. In all patients, PDGF median value was 8.96 pg/ml (IQR = 0–122.71 pg/ml). PDGF levels were not associated with patient’s age (Spearman’s *r* = 0.10, *p* = 0.366), disease duration (Spearman’s *r* = 0.13, *p* = 0.202), and baseline EDSS (Spearman’s *r* = 0.014, *p* = 0.897). At the time of hospitalization, 42 patients were in remitting phase and 58 patients presented with either clinical relapse or Gd+ lesions at MRI or both. No significant difference in CSF PDGF levels was found between patients with no activity (median = 12.89 pg/ml, IQR = 0–715.20 pg/ml) and patients with clinical and/or radiological activity at diagnosis (median = 6.19 pg/ml, IQR = 0–69.48 pg/ml; Mann–Whitney test *p* = 0.250). In addition, no significant difference was observed in PDGF values between RR (median = 7.19pg/ml, IQR = 0–61.9 pg/ml) and CIS patients (median = 9.98 pg/ml, IQR = 0–163.28 pg/ml; Mann–Whitney test *p* = 0.599). Analyzing prospective disease activity, patients without clinical relapse in the observational period showed higher PDGF levels (median = 19.39 pg/ml, IQR = 0–163.28 pg/ml) compared with patients presenting a clinical relapse (median = 0 pg/ml, IQR = 0–33.48 pg/ml; Mann–Whitney test *p* = 0.024). In addition, no significant association was observed between PDGF concentrations and MRI progression (Mann–Whitney test *p* = 0.796). Finally, no significant difference was found in PDGF concentration between patients with EDSS progression and patients without (Mann–Whitney test *p* = 0.415).

**Table 1:** Demographic and clinical characteristics of the whole sample of patients

		*N* = 100
Diagnosis, CIS	*n* (%)	31 (31%)
Diagnosis, RR	*n* (%)	69 (69%)
Age	Mean (SD)	33.7 (10.08)
Sex, female	*n* (%)	70 (70%)
Disease duration, months	Median (IQR)	8 (1.75–33.5)
Disease activity at diagnosis		
No activity	*n* (%)	42 (42%)
Clinical relapse and/or Gd+ MRI	*n* (%)	58 (58%)
Escalation to second line	*n* (%)	19 (19%)
EDSS at baseline	Median (IQR)	2 (1–2)

### CSF PDGF distribution and its categorization

Exploring the distribution of PDGF values, we observed a strong asymmetry and high variability. The mean value was 227.82 pg/ml with a SD of 879.98 pg/ml. Specifically, a leptokurtic (*k* = 76.34) and highly positively skewed (skewness coefficient = 8.30) distribution was observed. PDGF was not detected in 38% of patients. None of the transformation applied to obtain a better symmetrical distribution was useful. Therefore, to obtain a better data interpretation, we categorized the PDGF values according to the tertile (3 pg/ml and 54.28 pg/ml) splitting the data into three approximately equally sized group: “not detected PDGF” (*n* = 38); “medium PDGF,” PDGF values = 3–54.28 pg/ml (*n* = 29); and “high PDGF,” PDGF values > 54.28 pg/ml (*n* = 33).

No significant differences in age, gender, and diagnosis were found between the three groups. All patients received immunomodulatory treatment during the follow-up period. No significant differences both in the first-line treatment and in the escalation to second-line emerged between the three PDGF groups (Table 2).

**Table 2:** Clinical characteristics according to PDGF group

	*n*	PDGF group			*p*
		Absent	Medium	High	
		38	29	33	
Diagnosis, CIS	*n* (%)	11 (28.9%)	11 (37.9%)	9 (27.3%)	0.625
Diagnosis, RR	*n* (%)	27 (71.1%)	18 (62.1%)	24 (72.7%)	
Age	Mean (SD)	33.4 (9.38)	32.3 (8.98)	35.2 (11.82)	0.625^a^
Sex, F	*n* (%)	29 (76.3%)	18 (62.1%)	23 (69.7%)	0.458
Disease duration, months	Median (IQR)	8 (1.7–18.3)	7 (1–34)	12 (3–84)	0.218^b^
Disease activity at diagnosis					
No activity	*n* (%)	16 (42.1%)	11 (37.9%)	15 (45.5%)	0.817
Clinical relapse and/or Gd+ MRI	*n* (%)	22 (61.1%)	18 (62.1%)	18 (54.5%)	
Escalation to second line	*n* (%)	8 (21.1%)	7 (24.1%)	4 (12.1%)	0.446
EDSS at baseline	Median (IQR)	2 (1–2)	1.5 (1–2)	2 (1–2)	0.998

All *p* values refer to chi-square test

^a^Anova test

^b^Kruskal–Wallis test

### CSF PDGF levels and cytokines

To test whether CNS inflammation influences PDGF levels, we explored the correlation between CSF PDGF and the levels of the main pro- and anti-inflammatory molecules. Previous studies suggest that the concentrations of neurotrophins may be influenced by inflammation. In particular, immune cells from patients with RR multiple sclerosis secrete low levels of brain-derived neurotrophic factor (BDNF) [24] and IFN-β therapy increased BDNF levels [25]. Strong upregulation of PDGF was observed in peripheral blood leukocytes, with the highest expression after the disease maximum when reparative processes take place after acute inflammation [26].

We evaluated the correlation between PDGF levels and interleukin IL-12, GMCSF, FGF basic, GCSF, IL-10, IL-1-ra, IL-8, IL-6, IL-1β, IL-2, TNFα, and IFNγ. The analyses showed that PDGF positively correlated with IL-12 (Spearman’s *r* = 0.679, FDR-adjusted *p* < 0.001), IL-10 (Spearman’s *r* = 0.415, FDR-adjusted *p* < 0.001), GMCSF (Spearman’s *r* = 0.484, FDR-adjusted *p* < 0.001), and GCSF (Spearman *r* = 0.435, FDR-adjusted *p* < 0.001), as well as with FGF basic (Spearman’s *r* = 0.477, FDR-adjusted *p* < 0.001). A significant correlation was found with IL-1 receptor antagonist (Spearman’s *r* = 0.262, FDR-adjusted *p* = 0.021). The other associations were not significant (All FDR-adjusted *p* > 0.20).

### CSF PDGF groups and clinical disease activity

To explore whether CSF PDGF concentration could influence the clinical expression of brain damage, we examined clinical parameters of disease activity in the three PDGF groups.

**Fig. 1:**
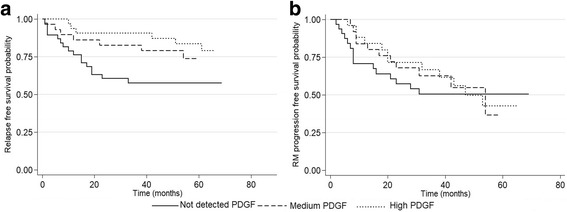
Influence of CSF PDGF levels on clinical and radiological disease activity. **a** The probability to not reach a clinical relapse, the RFS, according to the PDGF group. RFS was higher in both “medium PDGF” and “high PDGF” groups compared to patients with “not detected PDGF”. **b** The probability to not reach the first MRI progression during the observational period, the MRI progression-free survival in the three PDGF groups. No significant difference was found in the three PDGF groups

Overall, 29 patients had a clinical relapse during the observational period (42% in the “not detected PDGF”, 24% in the “medium PDGF,” and 18% in the “high PDGF”). The overall RFS was 69.5% (SE = 4.8%), and RFS mean time was 67.02 (SE = 3.35) months. The univariable Cox model showed a significant difference in RFS between the PGDF groups (*p* = 0.05) (Fig. 1a).

As PDGF levels significantly correlated with IL-12, GMCSF, FGF basic, GCSF, and IL-10, we evaluated whether these molecules may individually affect clinical disease activity. Setting a significance level of 0.10 (see “Statistical analysis” section), none of these molecules had a univariable significant effect on the RFS (Table 3). The final Cox regression model included PDGF groups, adjusting for patients’ escalation to second line and age. The adjusted effect of PDGF group was emphasized (*p* = 0.016), showing a protective effect on the RFS. In particular, RFS was higher in both “medium PDGF” (adj-HR = 0.28; 95% CI = 0.10–0.79; *p* = 0.017) and “high PDGF” groups (adj-HR = 0.33; 95% CI = 0.13–0.89; *p* = 0.029) compared to patients with “not detected PDGF” (Table 3).

**Table 3:** Uni- and multi-variate cox regression analysis of risk factors associated with relapse free survival

Univariable analysis	HR	95% CI	*p* value	Multivariable analysis	HR	95% CI	*p* value
Age, years	0.93	0.89–0.97	0.001	Age, years	0.93	0.89–0.97	0.002
Escalation to second line: no	1	Reference category		Escalation to second line: no	1	Reference category	
Yes vs no	3.31	1.56–7.04	0.002	Yes vs no	2.7	1.18–6.18	0.018
PDGF groups: not detected PDGF	1	Reference category		PDGF category: not detected PDGF	1	Reference category	
Medium PDGF vs not detected PDGF	0.5	0.20–1.21	0.124	Medium PDGF vs not detected PDGF	0.28	0.10–0.79	0.017
High PDGF vs not detected PDGF	0.36	0.14–0.92	0.033	PDGF vs not detected PDGF	0.33	0.13–0.89	0.029
MRI progression: no	1	Reference category		Not included in the multivariable final Cox regression model			
Yes vs no	2.19	1.03–4.64	0.041				
Sex: Male	1	Reference category					
Female vs male	1	0.46–2.20	0.996				
Disease duration, months	0.95	0.77–1.19	0.667				
IL-12	0.99	0.99–1.00	0.137				
GMCSF	1	0.99–1.01	0.908				
FGF basic	0.99	0.95–1.03	0.555				
GCSF	1.00	0.99–1.00	0.508				
IL-10	0.99	0.98–1.01	0.328				

No significant correlations were evidenced between CSF PDGF levels and other clinical parameters of disease activity. In particular, considering the total number of relapses during the observation time, no significant differences emerged between the three PDGF groups (Kruskal–Wallis test *p* = 0.459).

### CSF PDGF groups and radiological disease activity

It has been previously reported that different growth factors (i.e., BDNF) exert anti-inflammatory and anti-apoptotic effects in a murine model of MS (i.e., experimental autoimmune encephalomyelitis, EAE) [27]. To explore a possible immunomodulatory effect of PDGF, we first examined whether CSF PDGF levels at the time of diagnosis influence prospective radiological disease activity.

No significant differences emerged in the radiological parameters of disease activity between the three PDGF groups. In particular, the overall MRI progression-free mean time was 43.5 (SE = 3.01) months and the median MRI progression-free time was 53 months; the overall survival was 45.3% (SE = 6.7%) (Fig. 1b). The Cox model did not reveal significant differences between the three PDGF groups (*p* = 0.771). Other radiological measures of disease activity, including total number of active MRI, did not significantly differ between the three groups.

Indeed, MRI progression and clinical relapse were significantly associated (HR = 2.19, 95% CI = 1.03–4.64; *p* = 0.041). However, in the multivariable Cox model, the effect of MRI progression was no longer significant (HR = 1.13, 95% CI = 0.42–3.03; *p* = 0.801); therefore, on the basis of the forward procedure, it did not enter in the final equation model and the protective effect of PDGF group was not modified (*p* = 0.016). In addition, when we stratified for MRI progression (Yes/No), the effect of PDGF groups on the RFS remained significant (*p* = 0.027). The “medium PDGF” group as well as the “high PDGF” group had higher RFS compared with “not detected PDGF” (adj-HR = 0.30, 95% CI = 0.11–0.87; *p* = 0.027 and adj-HR = 0.35, 95% CI = 0.13–0.93; *p* = 0.035, respectively).

Model validation was evaluated through the measures described in the “Statistical analysis” section. In particular, the shrinkage coefficient was 83.9% indicating 16.1% lack of fit in the model, and the c index resulted equal to 0.73, showing good discrimination.

### CSF PDGF levels and disease progression

It has been demonstrated that PDGF exerts neuroprotective effects in MS reducing neurodegeneration. Therefore, we examined whether PDGF levels could influence clinical measures of disease progression, and no significant differences emerged between the three PDGF groups. In particular, both PI and MSSS did not significantly differ in the three PDGF groups (Kruskal–Wallis test *p* = 0.801 and *p* = 0.701, respectively).

We also explored whether clinical disability, as assessed by EDSS, could be influenced by PDGF. No significant differences were observed between the three groups in the baseline EDSS (see Table 2). In addition, EDSS at follow-up did not significantly differ between groups (all *p* > 0.08).

BREMS scores did not significantly differ between the three groups of patients (Kruskal–Wallis test *p* = 0.172).

### CSF PDGF levels and measures of neuronal damage

**Fig. 2:**
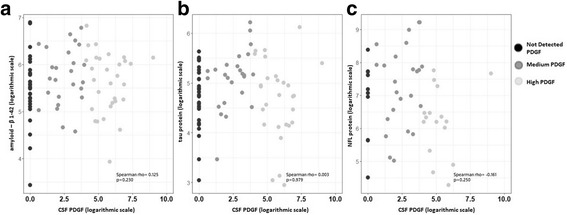
CSF PDGF levels and markers of neurodegeneration and axonal damage. The figure shows the correlations between CSF PDGF levels and the levels of amyloid-β 1–42 (**a**), tau protein (**b**), and NFL protein (**c**). To obtain a better graphical representation, all variables are depicted on logarithmic scale. Spearman rho correlations were calculated on the variables’ original scale. No significant correlation was found between CSF PDGF concentrations and the levels of markers of neurodegeneration and axonal damage. The *p* values showed were not adjusted by the Benjamini–Hochberg FDR controlling procedure

As previously argued, PDGF may contrast neuronal degeneration and damage promoting neuronal survival. To this aim, we explored the correlation between CSF PDGF levels and other established markers of neuronal and axonal damage. No significant correlation was found between CSF PDGF concentrations and the levels of amyloid-β 1–42 (Spearman’s *r* = 0.125, FDR-adjusted *p* = 0.383), tau protein (Spearman’s *r* = 0.003, FDR-adjusted *p* = 0.979), and NFL protein (Spearman’s *r* = − 0.161, FDR-adjusted *p* = 0.375; Fig. 2).

Therefore, to exclude the possibility that higher CSF PDGF levels could prevent a marked degree of structural damage after acute inflammation, we explored in a subgroup of 38 patients with radiological activity at the time of diagnosis the possible correlations between CSF PDGF levels (median = 20.98 pg/ml, IQR = 0–124.8 pg/ml) and markers of axonal damage. No significant correlation was found with CSF tau (Spearman’s *r* = 0.010, *p* = 0.953) and NFL proteins (Spearman’s *r* = − 0.301, *p* = 0.224).

## Discussion

Growth factors play numerous functions during embryogenesis and in different pathological conditions. PDGF consists of a family of five dimeric ligands (PDGF-AA, -AB, -BB, -CC, and -DD) interacting with two different receptors subtypes, as PDGF-alpha and beta [28, 29]. In MS, it has been proposed that PDGF may exert beneficial effects through different mechanisms, although the main pathophysiological processes are not completely identified.

In this study, we show that in a group of MS patients classified at the time of diagnosis as CIS and early RR-MS, higher CSF PDGF concentrations were associated with a beneficial effect on RFS, without affecting other clinical and radiological indexes of disease activity. In particular, as the number of patients with MRI progression in the observational period did not differ in the three groups, PDGF seems not to influence the aggressiveness of the disease. In addition, clinical disease activity is not influenced by PDGF levels as the total number of relapses during the observation time did not differ between the three PDGF groups. The finding that in our patients an anti-inflammatory milieu is associated to higher CSF PDGF levels is in line with the role of this neurotrophin in the recovery phases after acute inflammation. Indeed, the lack of negative correlation with pro-inflammatory cytokines could suggest that PDGF is not associated to a direct anti-inflammatory action and explain why disease activity is not influenced by PDGF levels. In particular, the beneficial effect on RFS is independent of first-line treatment and of indirect markers of disease activity such as escalation to second-line treatment, making unlikely the possibility that different disease course may explain our results. Indeed, our findings are in line with the hypothesis that PDGF expression during the recovery phases could promote clinical compensation of brain damage. Experimental evidence demonstrated that PDGF could enhance LTP induction, modulating the expression of genes involved in transcription-dependent synaptic plasticity [30]. The demonstration that LTP could be crucial for clinical recovery after acute brain lesion first came from preclinical studies [31]. It has been shown that in patients with RR-MS, higher CSF PDGF concentrations were associated to enhanced LTP-like plasticity [15, 16, 32] and better recovery after a relapse [16]. In addition, in patients presenting with Gd+ lesions on MRI, higher CSF PDGF levels were more frequently found in clinically silent patients compared with relapsing patients [15]. Overall, it is likely that LTP-like plasticity enhancement mediates the clinical beneficial effect of PDGF.

Members of the PDGF family have been associated to neuroprotective effects in different conditions, including excitotoxicity [33], energy deprivation and oxidative injury [34], toxicity associated to human immunodeficiency virus protein [30], and after brain ischemia [35]. To exclude the possibility that beneficial role of PDGF could be mediated by neuroprotective effects, we explored the influence of PDGF levels on measures of disease progression. As no significant correlation emerged with PI, MSSS, and EDSS progression, it is possible that PDGF concentrations at the time of diagnosis may not influence disease severity. We also explored whether PDGF concentrations influence the levels of some well-known biomarkers of neurodegeneration and neuronal damage such as amyloid-β, tau, and NFL proteins. CSF levels of amyloid-β and tau protein, a molecule involved in the stabilization of microtubules, were found altered in different neurodegenerative diseases [36]. Notably, in MS patients, altered amyloid-β metabolism was reported during acute inflammation [37]. Furthermore, NFL proteins are cytoskeleton components representing a marker of axonal damage in MS [38]. In MS, axonal damage is not only due to neurodegeneration but also to axonal transections occurring in acute demyelinating lesions [39]. Accordingly, increased CSF levels of NFL and tau proteins have been found in MS patients, and high CSF levels of axonal cytoskeletal proteins correlate with EDSS [40]. Axonal damage can follow acute demyelination as suggested by the findings that elevated CSF levels of NFL and tau proteins can be observed throughout the disease course and not confined to the late stages [41]. In our patients, no significant correlation emerged between PDGF and the CSF levels of amyloid-β, tau protein, and NFL protein. The lack of association between PDGF and the examined biomarkers suggests that the beneficial effect of PDGF may not be ascribed to a different degree of neuronal damage. It is worth noting that in our cohort of patients, CSF levels of biomarkers of neurodegeneration were assessed at the time of diagnosis; therefore, the lack of association could be due to the relatively short disease duration. As in the subgroup of patients with acute inflammation and radiological activity at the time of CSF withdrawal, no significant correlation was found between PDGF levels and tau protein and NFL protein; it seems unlikely that PDGF levels in the post-acute phases may influence the subsequent building up of axonal damage. Other limitations of the present study include the lack of prospective radiological measures of neurodegeneration (i.e., gray matter cortical atrophy); nevertheless, the lack of difference in disease severity between the three PDGF groups makes unlikely the possibility that different degree of neurodegeneration may explain the results.

## Conclusions

Our findings suggest that PDGF could promote a prolonged relapse-free period, without influencing inflammatory reactivation and long-term disability. These results agree with the hypothesis that PDGF could specifically enhance the clinical compensation of brain damage, in line with the specific role of PDGF during recovery phases in MS. Accordingly, in EAE, PDGF is strongly upregulated particularly after the peak of disease [26]. In our sample of patients, high PDGF levels are associated with high levels of anti-inflammatory cytokines and other neurotrophic factors. As the exact role of these molecules in MS pathophysiology is still scarcely defined, further studies are required to clarify the specific role of the different anti-inflammatory molecules. However, it should be remarked that in our patients, only PDGF levels were associated with a prolonged RFS. Overall, it is likely that the chance to develop an effective anti-inflammatory response in the post-acute phases influences both the immediate recovery and the mid-term clinical course. This could suggest that a successful clinical recovery after a relapse should be characterized by a shift toward an anti-inflammatory response promoting the expression of key molecules able to favor LTP.

Notably, the beneficial effects of PDGF may be related to the convergence existing between the intracellular signaling pathways involved in LTP and neuronal survival [38]. Accordingly, CSF PDGF levels are reduced in progressive MS phenotypes characterized by both increased neurodegeneration [42] and absent LTP-like plasticity [15]. Therefore, the beneficial clinical effects of PDGF could be particularly relevant in the early phases of MS, when the clinico-radiological paradox is more frequently observed.

## Abbreviations

BDNF: Brain-derived neurotrophic factor
BREMS: Bayesian risk estimate for multiple sclerosis
CIS: Clinically isolated syndrome
CNS: Central nervous system
DMT: Disease-modifying therapy
EAE: Experimental autoimmune encephalomyelitis
EDSS: Expanded disability status scale
ELISA: Enzyme-linked immunosorbent assay
FDR: False discovery rate
FGF: Fibroblast growth factor
GCSF: Granulocyte colony-stimulating factor
GMCSF: Granulocyte macrophage colony-stimulating factor
HR: Hazard ratio
IFN: Interferon
IL: Interleukin
IQR: Interquartile range
LTP: Long-term potentiation
MRI: Magnetic resonance imaging
MS: Multiple sclerosis
MSSS: Multiple sclerosis severity scale
NFL: Neurofilament light
PDGF: Platelet-derived growth factor
PI: Progression index
RFS: Relapse-free survival
RR: Relapsing-remitting
